# Preoperative Molecular Markers in Thyroid Nodules

**DOI:** 10.3389/fendo.2018.00179

**Published:** 2018-04-18

**Authors:** Zeyad T. Sahli, Philip W. Smith, Christopher B. Umbricht, Martha A. Zeiger

**Affiliations:** ^1^Department of Surgery, The Johns Hopkins University School of Medicine, Baltimore, MD, United States; ^2^Department of Surgery, University of Virginia, Charlottesville, VA, United States

**Keywords:** thyroid cancer, non-invasive follicular thyroid neoplasm with papillary-like nuclear features, molecular test, Afirma, Thyroseq

## Abstract

The need for distinguishing benign from malignant thyroid nodules has led to the pursuit of differentiating molecular markers. The most common molecular tests in clinical use are Afirma^®^ Gene Expression Classifier (GEC) and Thyroseq^®^ V2. Despite the rapidly developing field of molecular markers, several limitations exist. These challenges include the recent introduction of the histopathological diagnosis “Non-Invasive Follicular Thyroid neoplasm with Papillary-like nuclear features”, the correlation of genetic mutations within both benign and malignant pathologic diagnoses, the lack of follow-up of molecular marker negative nodules, and the cost-effectiveness of molecular markers. In this manuscript, we review the current published literature surrounding the diagnostic value of Afirma^®^ GEC and Thyroseq^®^ V2. Among Afirma^®^ GEC studies, sensitivity (Se), specificity (Sp), positive predictive value (PPV), and negative predictive value (NPV) ranged from 75 to 100%, 5 to 53%, 13 to 100%, and 20 to 100%, respectively. Among Thyroseq^®^ V2 studies, Se, Sp, PPV, and NPV ranged from 40 to 100%, 56 to 93%, 13 to 90%, and 48 to 97%, respectively. We also discuss current challenges to Afirma^®^ GEC and Thyroseq^®^ V2 utility and clinical application, and preview the future directions of these rapidly developing technologies.

## Introduction

Thyroid nodules are common among adults over the age of 60 years, with a prevalence of 50–70% (1, 2). Moreover, the incidence of thyroid cancer in the United States has increased by 211% between 1975 and 2013 (3), due to both an improved detection of small (<2 cm) thyroid nodules by thyroid ultrasonography and a true increase in thyroid cancer incidence (4). Nevertheless, the vast majority (85–95%) of thyroid nodules are benign (5). For this reason, the ability to distinguish between benign and malignant nodules is important in order to spare patients unnecessary diagnostic surgery.

Fine needle aspiration (FNA) to facilitate this distinction first became widely practiced in the early 1980s (6), and is widely recognized as the gold standard initial diagnostic procedure in the differential diagnosis of thyroid nodules, being accurate, safe, and cost effective (7, 8). The sensitivity (Se), specificity (Sp), positive predictive value (PPV), negative predictive value (NPV), false-negative (FN) rate, and false-positive (FP) ranges of an FNA are 88.2–97.0%, 47.0–98.2%, 52.0–98.0%, 89.0–96.3%, 0.5–10.0%, and 1.0–7.0%, respectively (9).

In 2009, the Bethesda classification system for FNA reporting was introduced by the National Cancer Institute, and recently, revised, included six categories based upon cytopathological features, with an associated malignancy rate and standardized management recommendation for each category (Table 1) (10). FNA reliably establishes the diagnosis of a benign or malignant nodule in 70–80% of all cases (11) and has decreased the proportion of benign nodules unnecessarily resected from 86 to 50% (12). However, 20–30% of FNA cases have indeterminate or suspicious cytological results that include Bethesda III, IV, and V categories (12) and, of these, 6–75% are malignant on final surgical pathology (13, 14). Due to the uncertainty of malignancy in these patients, their management has been challenging, usually including a repeat FNA or a diagnostic lobectomy. For this reason, the need for distinguishing benign from malignant lesions in this subset of thyroid nodules has led to the pursuit of differentiating molecular markers.

**Table 1:** The Bethesda system for reporting thyroid cytopathology (10).

Bethesda group	Diagnostic category	Abbreviation	Malignancy rate	
			Before NIFTP reclassification (NIFTP malignant)	After NIFTP reclassification (NIFTP benign)
I	Non-diagnostic/unsatisfactory	–	5–10%	No change
II	Benign	B	0–3%	No change
III	Atypia of undetermined significance/follicular lesion of undetermined significance	AUS/FLUS	10–30%	6–18%
IV	Follicular neoplasm/suspicious for follicular neoplasm	FN/SFN	25–40%	10–40%
V	Suspicious for malignancy	SM	50–75%	45–60%
VI	Malignant	M	97–99%	94–96%

Interest in achieving this distinction increased in 2002 with the recognition of the oncogenic role of the *BRAF V600E* mutation in approximately 58–69% of papillary thyroid cancers (PTC) (15, 16). However, genetic testing for *BRAF V600E* alone for the detection of PTCs is inadequate for clinical decision making due to its low sensitivity of 60% for PTC (17). Indeed, our group first published its use in indeterminate and suspicious thyroid lesions and found it to add minimal clinical value (18). In addition to studying the diagnostic utility of *BRAF V600E*, numerous studies have investigated the association of *BRAF V600E* and patient prognosis. However, the correlation between *BRAF V600E* and clinical features of PTCs has yielded inconsistent results. Some studies report that *BRAF V600E* is associated with a more advanced phenotype including an increased risk of lymph node metastasis, cancer recurrence, and patient mortality (19–21), while others report no such associations (22). Moreover, thyroid cancer with *BRAF V600E* and *TERT* promoter mutations has been associated with worse clinico-pathological outcomes (23, 24). *BRAF* testing can also be useful in deciding treatment in the setting of known metastatic thyroid cancer. Direct tyrosine kinase inhibitors, such as vemurafenib (25), dabrafenib (26), and sorafenib (27), have been shown to be effective in *BRAF V600E* metastatic thyroid cancers.

Since mutational analysis of single genes has not proven adequate in guiding management decisions in indeterminate or suspicious thyroid nodules, attention turned to using panels of molecular markers. Currently, the most common molecular tests in clinical use are Afirma^®^ Gene Expression Classifier (GEC) and Thyroseq^®^ V2 (28). Introduced in 2011 by Veracyte, the Afirma^®^ GEC has been considered a “rule-out” malignancy test. It includes a 142-gene expression molecular assay and uses microarray technology to measure the mRNA expression profiles to determine whether a thyroid nodule is “suspicious” or “benign.” The test’s primary aim is to spare patients with cytologically indeterminate FNA samples unnecessary diagnostic surgery (29). Among indeterminate/suspicious nodules (Bethesda Type III–V), the test has both a high Se (92%) and high NPV (93%) (30) (Table 2). In contrast, it has a low Sp (52%) and PPV (47%), and cannot accurately identify malignant lesions alone.

**Table 2:** Afirma Gene Expression Classifier (GEC) literature review.

Author	Year	Study type	Follow-up period	Bethesda category	Sample size	GEC suspicious	Total surgery	Malignant nodules	NIFTP	Diagnostic value			
										Se (%)	Sp (%)	PPV (%)	NPV (%)
Afirma^®^ GEC (30)	2012	Prospective, blinded multicenter	301 days (Median)	Total	265	165	265	85	–	92	52	47	93
				III	129	74	129	31		90	53	38	95
				IV	81	49	81	20		90	49	37	94
				V	55	42	55	34		94	52	76	85

Hang et al. (31)	2017	Retrospective, single institution	–	III	133	112	133	27	21	100	24	42	100
				IV	42	39	42	6	4	90	6	23	67

Harrison et al. (32)	2017	Retrospective, single institution	–	III	100	–	37	13	–	–	–	35	–
				IV	10	–	4	1		–	–	25	–

Kay-Rivest et al. (33)	2017	Retrospective, multicenter	–	III, IV	172	83	77	38	–	–	–	49	–

Samulski et al. (34)	2016	Retrospective, single institution		Total	294	133	128	33	11	93	17	39	81
			–	III	166	60	60	15	5	95	19	40	88
				IV	122	73	68	18	6	92	15	39	75

Wu et al. (35)	2016	Prospective, single institution	–	Total	245	132	128	63	–	94	31	57	83
				III	217	115	107	55		93	29	58	79
				IV	28	17	21	8		100	38	50	100

Yang et al. (36)	2016	Retrospective, single institution	–	Total	189	94	67	32	–	100	15	51	100
				III	165	81	55	26		100	7	49	100
				IV	24	13	12	6		100	50	67	100

Chaudhary et al. (37)	2016	Retrospective, single institution	–	Total	158	85	73	28	–	100	15	100	20
				III	89	41	45	8		100	18	20	100
				IV	69	44	41	21		100	11	55	100

Abeykoon et al. (38)	2016	Retrospective, single institution	–	III, IV	34	17	16	12	–	–	–	49	–

Noureldine et al. (39)	2016	Prospective, single institution	–	III, IV	99	89	89	37	–	97	9	42	83

Al-Qurayshi et al. (40)	2016	Retrospective, single institution	–	Total	154	96	112	50	–	78	40	51	69
				III	114	66	84	36		78	52	55	76
				IV	40	30	28	14		79	0	44	0

Witt (41)	2016	Retrospective, single institution		III, IV	47	15	15	6	–	–	–	40	–

Wong et al. (42)	2016	Retrospective, single institution	–	III, IV	63	63	63	8	14	–	–	35	–

Zhu et al. (43)	2015	Retrospective, single institution	–	III, IV	45	21	10	6	–	–	–	60	–

Celik et al. (44)	2015	Retrospective, single institution		Total	40	23	20	10	–	100	20	56	100
			–	III	11	6	6	4		100	0	67	Null
				IV	29	17	14	6		100	25	50	100

Marti et al. (45)	2015	Retrospective, multicenter	–	III: 103 IV: 62	165	104	70	27	–	100	16	43	100

Brauner et al. (46)	2015	Retrospective, multicenter	–	III, IV, and Hurthle Cell	72	45	47	6	–	100	8	14	100

McIver et al. (47)	2014	Prospective, single institution	9.5 months	Total	72	44	36	6	–	83	10	16	75
				III	9	–	5	1		100	0	20	Null
				IV	63	–	31	5		80	12	15	75

Lastra et al. (48)	2014	Retrospective, single institution	–	Total	132	62	50	22	–	100	7	54	100
				III	68	23	18	11		100	0	61	Null
				IV	64	39	32	11		100	10	37	100

Aragon Han et al. (49)	2014	Retrospective, single institution	–	III, IV	37	36	37	16	–	100	5	44	100

Alexander et al. (50)	2013	Retrospective, multicenter	8.5 months	Total	339	148	132	54*	–	98	13	44	91
				III	165	66	48	23		–	–	–	–
				IV	79	73	65	24		–	–	–	–
				V	13	9	8	6		–	–	–	–

Harrell et al. (51)	2013	Retrospective, single institution		Total	58	36	35	18	–	94	24	57	80
			–	III	–	22	–	–		100	33	64	100
				IV	–	8	–	–		75	0	38	0

ThyroSeq^®^ v2, introduced in 2014 by CBL Path, is designed to identify malignant thyroid nodules by next generation sequencing (NGS), detecting 14 thyroid cancer-related genetic mutations, including *RAS* and *BRAF* mutations, 42 types of gene fusions associated with thyroid cancer, including *PAX*8/*PPARγ* and *RET/PTC* rearrangements, and mRNA expression levels for 16 genes; it is therefore considered a “rule-in” malignancy test (29). Among Bethesda Type III – IV nodules, the test is marketed as having a high Se, Sp, PPV, and NPV of 90–91%, 92–93%, 77–83%, and 96–97%, respectively, as well as having the ability to stratify risk based on the mutation detected (52, 53) (Table 3).

**Table 3:** Thyroseq V2 literature review.

Author	Year	Study type	Follow-up period	Bethesda category	Sample size	Thyroseq suspicious	Total surgery	Malignant nodules	NIFT-P	Diagnostic value			
										Se (%)	Sp (%)	PPV (%)	NPV (%)
ThyroSeq^®^ v2 (53)	2015	Retrospective, Single Institution	–	III	465	31	95	22	–	91	92	77	97

ThyroSeq^®^ v2 (52)	2014	Single Institution	–	IV	143	42	143	39	–	90	93	83	96

Taye et al. (54)	2017	Prospective, Multicenter	–	III, IV	156	51	63	10	3	91	45	27	96

Valderrabano et al. (55)	2017	Retrospective, Single Institution	–	Total	190	45	102	15	5	70	77	42	91
				III	104	22	52	5	2	43	71	19	89
				IV	86	23	50	10	3	85	84	65	94

Shrestha et al. (56)	2016	Retrospective, Single Institution	–	Total	39	23	39	14	–	93	60	57	94
				III	27	15	27	9		89	61	53	92
				IV	12	8	12	5		100	57	63	100

Khatami et al. (57)	2016	Retrospective, Single Institution	3–6 months	III, IV, V	42	7	7	4	–	–	–	57	–

Toraldo et al. (58)	2016	Prospective, Single Institution	–	III, IV	148	51	45	20	–	95	60	66	94

Shrestha et al. (59)	2016	Retrospective, Single Institution	–	III	41	–	41	–	–	83	62	90	48
				IV	14	–	14	–	–	80	56	50	90

In this manuscript, we review the current published literature surrounding Afirma^®^ GEC and Thyroseq^®^ V2, discuss current challenges to their utility; and clinical application, and preview the future directions of these rapidly developing technologies.

## Clinical Management of Indeterminate Thyroid Nodules

The 2015 American Thyroid Association (ATA) (60) and the 2016 American Association of Clinical Endocrinologists (AACE) (9) clinical guidelines recommend “considering,” molecular testing for indeterminate nodules. If molecular testing is being considered, ATA recommends that patients “should be counseled regarding the potential benefits and limitations of testing and about the possible uncertainties in the therapeutic and long-term clinical implications of results” (strong recommendation, low-quality evidence) (60). However, long-term outcome data on the use of molecular markers for therapeutic decision-making is currently unavailable. A recent report estimated that standard application of the GEC for all indeterminate thyroid nodules would result in only a 7.2% decrease in thyroidectomy volume (61). Similarly, two studies by our group showed that molecular markers did not significantly affect the surgical decision-making process, where only 7.9–8.4% of patients had altered clinical management as a result of molecular testing (39, 62). For these reasons, patient benefit from molecular marker use in routine clinical practice is likely marginal. Moreover, the AACE 2016 guidelines recommend molecular testing to complement cytologic evaluation in indeterminate nodules (Grade A recommendation), but only when the “results are expected to influence clinical management” (Grade A recommendation) (9). Testing for detection of *BRAF, RET/PTC, PAX8/PPRG*, and *RAS* mutations can be considered (Grade B recommendation). Furthermore, with the exception of *BRAF V600E*, there is insufficient evidence “to recommend in favor of or against the use of mutation testing as a guide to determine the extent of surgery” (Grade A recommendation) (9).

Importantly, molecular testing is not recommended in patients with an indeterminate thyroid nodule if other indications for surgery are present such as a nodule greater than 4 cm, compressive symptoms, or personal preference (63). The utility of molecular testing in Bethesda Type V nodules at institutions with a high prevalence of malignancy is low, and provides little additional benefit from a “positive” test result due to the similar PPV as that of a Bethesda Type V FNA result. Moreover, a diagnostic lobectomy would still be recommended in the case of a “negative” result. Finally, a limitation of the current molecular markers is their insufficient data to recommend use among pediatric patients (≤18 years) (64). Until these tests can be validated using this patient population, they cannot be routinely used to complement the indeterminate FNA cytology results.

## Current Published Literature

Current published literature regarding Afirma^®^ and Thyroseq^®^ V2 validation studies are summarized in Tables 2 and 3. The data were summarized from the results of a PubMed search for English language studies that reported diagnostic accuracy in observational clinical settings published for GEC and Thyroseq^®^ V2 up to November 30, 2017. References from the retrieved articles were also searched for additional studies. Inclusion criteria included reporting molecular marker diagnostic accuracy or enough information to calculate sensitivity, specificity, NPV, and PPV among Bethesda Type III or IV lesions. All calculations were made using the available published information. To adhere to current clinical guidelines, non-invasive follicular thyroid neoplasm with papillary-like nuclear features (NIFTP) and malignant pathologies were categorized as malignant or “requiring resection.” Of the published literature, only two studies included pediatric patients in their cohort (31, 32).

Among Afirma^®^ GEC studies, Se, Sp, PPV, and NPV ranged from 75 to 100%, 5 to 53%, 13 to 100%, and 20 to 100%, respectively. Among Thyroseq^®^ V2 studies, Se, Sp, PPV, and NPV ranged from 40 to 100%, 56 to 93%, 13 to 90%, and 48 to 97%, respectively. Valderrabano et al. report that the wide variation among reported diagnostic values can be explained by different defining characteristics of the study populations such as institutional prevalence of malignancy sample size, Bethesda Type included or combination thereof used, the proportions of each Bethesda Type, the definition of “benign” used in the study, and Hürthle cell (HC) predominance (65). Furthermore, among post-validation studies, the molecular test outcome itself influenced the clinical management.

Supporting this, numerous studies have reported a lower specificity or higher false positive rate in GEC tests among indeterminate nodules with HC predominance. Brauner et al. reported 86% (37/43) patients with a GEC suspicious result had unnecessary surgery (46). The authors include a grouped cohort analysis of 122 HC predominant nodules between 2012 and 2014, showing 85 of 95 (89.5%) benign pathologies identified as GEC suspicious (46). Another study by Parajuli et al. reported on GEC’s increase in false positive rate among HC predominant nodules, but did not observe the same increase in Thyroseq^®^ V2 (66). Additional studies assessing Thyroseq^®^’s performance in HC predominant nodules are lacking.

One component of GEC, the Medullary Thyroid Cancer (MTC) Classifier, has been far less studied (Table 4). Among the few existing studies, Se, Sp, PPV, and NPV ranged from 91 to 100%, 100%, 98 to 100%, and 99 to 100%, respectively. Further evaluation of the MTC Classifier is needed to establish its clinical efficacy.

**Table 4:** Medullary thyroid cancer (MTC) classifier and diagnostic value.

Author	Year	Study type	Follow-up period	Bethesda category	Sample size	MTC suspicious	Surgery	MTC prev.	Diagnostic value			
									Se (%)	Sp (%)	PPV (%)	NPV (%)
Pankratz (67)	2016	Retrospective, single institution	–	–	27	26	27	27	96	–	–	–
Kloos (68)	2016	Prospective, blinded multicenter	–	III–VI	10,488	43	43	42	–	–	98	–
Alexander et al. (30)	2012	Prospective, blinded multicenter	301 days (median)	III–VI	441	4	441	4	100	100	100	100
Training set (69)	2010	Prospective, blinded multicenter	–	–	220	22	220	20	91	100	100	99
Chudova et al. (69)	2010	Prospective, blinded multicenter	–	I–VI	48	0	48	0	–	100	–	99

## Current Challenges of Molecular Marker Assays

Current challenges to the application of molecular markers are fourfold: (A) the recent introduction of the histopathological diagnosis NIFTP, (B) the correlation of genetic mutations within both benign and malignant pathologic diagnoses, (C) the lack of follow-up of molecular marker negative nodules, and (D) the cost-effectiveness of molecular markers.

### Non-Invasive Follicular Thyroid Neoplasm With Papillary-Like Nuclear Features

In March 2015, Nikiforov et al. introduced the new histopathological term NIFTP, previously known as encapsulated follicular variant of papillary thyroid cancer, representing an indolent entity with very low risk of recurrence (70). Major diagnostic characteristics of NIFTP include features of FVPTC, such as a follicular growth pattern and nuclear features of PTC (enlargement, crowding, elongation, irregular contours, grooves, pseudoinclusions, and chromatin clearing), but a lack of vascular or capsular invasion, key features differentiating NIFTP from FVPTC.

This new diagnosis represents a dramatic shift in thyroid pathology where an estimated 61% of lesions previously classified as FVPTCs will now be classified as NIFTP, thus decreasing the percentage of “malignancies” on final pathology compared with FNA. On pre-operative cytology, NIFTP is associated with FNA Bethesda Category III, IV, V, or VI in 15, 56, 27, and 2% of tumor samples, respectively (71). As a consequence, this has created a shift in the malignancy rate associated with each Bethesda category. Strickland et al. evaluated a cohort of 655 FNAs with subsequent resection specimens (72). When taking into account the new NIFTP diagnosis, indeterminate, and suspicious FNA samples of Bethesda III, IV, and V had an absolute decrease in rate of malignancy by 17.6, 8.0, and 41.5%, respectively (72). Similarly, Faquin et al. reported an absolute decrease in rate of malignancy in Bethesda III, IV, and V by 13.6, 15.1, and 23.4%, respectively (73).

Despite NIFTP’s extremely low-recurrence rate of 0.6% (two cases), there remains disagreement regarding NIFTP’s true malignant potential (74–80). Despite its likely benign and, at worst, indolent nature, current ATA guidelines recommend lobectomy as definitive therapy for NIFTP. More importantly, however, and, apropos of this review, Afirma^®^ GEC and Thyroseq^®^ V2 validation studies occurred before the establishment of NIFTP as a distinct entity. Because of this one needs to be circumspect about the real utility of these marker panels. And, as a consequence, these molecular diagnostic panels require recalibration to appropriately account for the newly introduced entity, NIFTP; a lesion that should likely not be considered malignant (70, 81, 82).

### Correlating Mutations to Pathology

The correlation between presence of mutations and malignancy is imprecise. Among 967 Bethesda Type III, IV, and V nodules, the detection of any mutation conferred the risk of histologic malignancy of 88, 87, and 95%, respectively (83). However, even in nodules with no detected mutations, the malignancy rates were 6, 14, and 28%, respectively. A systematic review by our group included 8,162 patients, of whom 42.5% had benign lesions (84). Among the benign lesions, *RAS* mutations, *RET/PTC* rearrangements, and *PAX8/PPAR-gamma* rearrangements were present up to 48, 68, 55% of the time, respectively. Thus, benign nodules frequently harbor mutations, while some malignant lesions harbor no detected mutations. The combination of the variable and potentially high level of mutations among benign nodules may explain the low specificity and PPV seen in Afirma. Furthermore, their prominence in benign lesions may also challenge the reported PPV of Thyroseq V2.

This issue is further complicated when an indolent tumor, such as NIFTP, should be resected according to current ATA guidelines. NIFTP is commonly associated with *RAS* mutations (8/27, 29.6%) and its diagnosis is incompatible with the presence of *BRAF V600E* mutations (70, 79). Moreover, Nikiforov et al. describe that 22% (6/27) of NIFTP samples harbor no detectable mutations. To conform to the recommendation that this indolent lesion be resected, new validation studies must show the reliable identification of NIFTP by molecular markers, an unlikely occurrence given the fact that benign lesions also harbor them.

### Molecular Marker Negative Nodule Follow-Up

Despite a number of studies exploring the diagnostic value of GEC and Thyroseq^®^ V2, the current published literature includes discrepancies in the follow-up of molecular marker negative nodules and their consideration as a benign pathology (85, 86). Consequently, this may lead to inaccuracies in diagnostic value calculation. A systematic review by Duh et al. (85) highlighted these issues. They included 12 studies and discussed the exclusion of cytologically indeterminate, GEC benign nodules from diagnostic performance calculations (malignant versus benign), leading to an erroneous decrease in Sp and NPV. This is due to the lack of surgical pathology specimens to establish a definitive diagnosis, as well as a lack of follow-up of GEC benign nodules to establish a reference diagnosis. To establish a diagnostic “reference standard” in these nodules that have not undergone surgery and include them in calculations, the authors argue that they should be considered as “true negative” only if no suspicious changes are noted on scheduled interval ultrasound examinations. However, even the natural history of benign thyroid nodules has been described to involve size changes. Indeed, a 5-year prospective study involving 1,567 sonographically or cytologically benign thyroid nodules showed nodule growth in 11.1% (87). However, thyroid cancer was diagnosed in five original nodules (0.3%), of which, only two had an increase in size. Furthermore, a retrospective study ranging from 1 month to 5 years, reported that 39% of the 268 benign thyroid nodules showed at least a 15% change in nodule volume (88). Only one of the 74 repeat-FNAs was malignant. The authors conclude that an increase in nodule volume alone is not a reliable predictor of malignancy.

Two studies have described their experience with follow-up of GEC benign nodules on ultrasound. A study by Angel et al. including 56 patients with cytologically indeterminate, GEC benign nodules followed for a median of 13 months exhibited similar growth (≥20% in two dimensions or ≥50% in volume) to cytologically benign nodules (86). Furthermore, in a grouped cohort analysis by Kloos et al. of 443 GEC benign patients in six studies with a reported follow-up time of 7–26 months, 380 patients (85.8%) were spared unnecessary surgery (89). Clearly, the currently available follow-up periods are inadequate for a definitive assessment, and larger, prospective studies are needed to further evaluate the behavior of cytologically indeterminate, molecular marker “negative” thyroid nodules to help guide recommendations for management.

### Cost-Effectiveness

The cost-effectiveness of GEC and Thyroseq^®^ has also been an intense area of research. The cost for GEC and MTC is $4,875 while the cost for Afirma^®^ MTC alone is $975 and that of Afirma^®^ BRAF is $475 (Table 5). The cost of Thyroseq^®^ V2 is $3200 (29). Despite these high costs, insured patient costs are capped at $300 for either GEC or ThyroSeq^®^ V2. Numerous studies have reported on the cost-effectiveness of both GEC (90, 91) and Thyroseq^®^ V2 (92).

**Table 5:** Molecular marker cost.

	Afirma^**®**^	ThyroSeq V2
Cost	$4,875 (Afirma^®^ GEC & MTC)	$3,200
	$975 (Afirma^®^ MTC)	
	$475 (Afirma^®^ BRAF)	

Patient insurance coverage	$300 (Afirma^®^ GEC & MTC)	$300
	$80 (Afirma^®^ MTC)	
	$50 (Afirma^®^ BRAF)	

Estimated cost-effectiveness	Standard of care $12,172 versus GEC $10,719 (91)	Standard of care $11,505 versus Thyroseq^®^ cost $7,683 (92)
	Standard of care $11,505 versus GEC $13,027 (92)	

A 5-year cost effectiveness study of routine use of GEC reported 74% fewer operations for benign nodules with no increase in untreated cancers. Compared with standard clinical management based only on indeterminate FNA results, GEC may lower overall costs (standard cost $12,172 versus GEC cost $10,719) and improve quality of life for patients (91). Another study reported that to be cost effective, GEC’s specificity would have to be greater than 68% and decrease the number of unnecessary surgeries performed on benign nodules by more 50% (90). However, a study by Yip et al. compared the average cost per patient with Bethesda IV nodules larger than 1 cm extending 10 years from follicular neoplasm diagnosis in three groups: standard of care, GEC, and Thyroseq^®^. The authors reported a 13% increase in average cost per patient when using GEC at $13,027 (range $12,373–$13,666) when compared with the standard of care $11,505 (range $10,676–$12,347), but a 30% reduction in those using Thyroseq^®^ cost $7,683 (range $7,174–$8,333) (92).

Despite the conflicting results of GEC cost-effectiveness studies and the paucity of analyses on Thyroseq^®^, these studies highlight the need to closely examine cost-effectiveness with the use of genetic studies. However, the true impact of thyroid molecular tests on a population’s health care costs can only be determined taking cancer prevalence into account. Furthermore, cost-effectiveness also depends on proper education to ensure that these tests are only used in indicated clinical settings.

## Conclusion and Future Direction

Molecular markers are a rapidly developing field despite the current limitations. Veracyte and CBL Path have begun to address the challenges discussed here. In 2017, Veracyte launched the Afirma^®^ Genomic Sequencing Classifier (GSC), its newest product which tests a total of 10,196 genes. The Afirma^®^ GSC test system is composed of a series of classifiers including parathyroid (mRNA expression), MTC (mRNA expression), *BRAF* (mRNA expression + variants), and *RET/PTC* fusion (fusion transcripts). GSC also includes a follicular content index (mRNA expression), HC index (mRNA expression and mitochondrial transcripts), and Hürthle neoplasm index (mRNA expression and chromosomal level loss of heterozygosity). The reliance on mRNA could prevent detection of mutations undetectable in transcriptome-based assays, such as telomerase reverse transcriptase (hTERT) promoter mutations. Veracyte states that GSC addresses the weaknesses of GEC by significantly increasing molecular test specificity, using a validation cohort with 15 NIFTP samples, and improving the diagnostic performance among HC lesions. Among Bethesda Type III and IV nodules, Veracyte quotes a Se, Sp, NPV, and PPV of 91, 68, 96, and 50%, respectively, when compared with the GEC parameters of 89 50, 93, and 46%, respectively. Among HC lesions, Sp has increased from 11.8 to 58.8%.

Similarly, in 2017 CBL Path sought to address similar issues with the release of Thyroseq^®^ V3 (93). It has an expanded its assay from 56 to 112 genes, detecting mutations, gene fusions, gene expression alterations, and copy number variations. In a prospective double-blind multicenter study using 257 nodules with Bethesda Types III–V (including 11 NIFTP samples, 10 HC carcinomas, 34 HC adenomas, and 5 hyperplastic nodules with HC predominance), Thyroseq^®^ V3 is reported to have an increased diagnostic value, including a Se of 98% (from 96.9%) and specificity of 81.8% (from 74.0%). Moreover, HC samples had an Se and Sp of 100.0 and 66.7%, respectively.

As experience accumulates with these next generation tests, we will gain a better understanding of how well they mitigate the limitations and challenges addressed herein. As our understanding of genetic drivers of malignant cancers and our understanding of NIFTP’s malignant potential becomes clearer, molecular markers will continue to more accurately identify malignant nodules as well as to spare patients from unnecessary surgery.

## Author Contributions

ZS conducted the literature review. ZS, PS, and CU drafted and revised the manuscript. MZ drafted and revised the manuscript and, as the senior and corresponding author, takes full responsibility.

## Conflict of Interest Statement

The authors declare that the research was conducted in the absence of any commercial or financial relationships that could be construed as a potential conflict of interest.
